# Fractal Dimension Analysis of Subcortical Gray Matter Structures in Schizophrenia

**DOI:** 10.1371/journal.pone.0155415

**Published:** 2016-05-13

**Authors:** Guihu Zhao, Kristina Denisova, Pejman Sehatpour, Jun Long, Weihua Gui, Jianping Qiao, Daniel C. Javitt, Zhishun Wang

**Affiliations:** 1 School of Information Science and Engineering, Central South University, Changsha 410083, China; 2 Department of Psychiatry, Columbia University College of Physicians and Surgeons, New York, NY 10032, United States of America; 3 Sackler Institute for Psychobiology, Columbia University College of Physicians and Surgeons, New York, NY 10032, United States of America; 4 Division of Developmental Neuroscience, New York State Psychiatric Institute, New York, NY 10032, United States of America; 5 Nathan S. Kline Institute for Psychiatric Research, Orangeburg, NY 10962, United States of America; 6 College of Physics and Electronics, Shandong Normal University, Jinan 250014, China; University Of Cambridge, UNITED KINGDOM

## Abstract

A failure of adaptive inference—misinterpreting available sensory information for appropriate perception and action—is at the heart of clinical manifestations of schizophrenia, implicating key subcortical structures in the brain including the hippocampus. We used high-resolution, three-dimensional (3D) fractal geometry analysis to study subtle and potentially biologically relevant structural alterations (in the geometry of protrusions, gyri and indentations, sulci) in subcortical gray matter (GM) in patients with schizophrenia relative to healthy individuals. In particular, we focus on utilizing Fractal Dimension (FD), a compact shape descriptor that can be computed using inputs with irregular (i.e., not necessarily smooth) surfaces in order to quantify complexity (of geometrical properties and configurations of structures across spatial scales) of subcortical GM in this disorder. Probabilistic (entropy-based) information FD was computed based on the box-counting approach for each of the seven subcortical structures, bilaterally, as well as the brainstem from high-resolution magnetic resonance (MR) images in chronic patients with schizophrenia (n = 19) and age-matched healthy controls (n = 19) (age ranges: patients, 22.7–54.3 and healthy controls, 24.9–51.6 years old). We found a significant reduction of FD in the left hippocampus (median: 2.1460, range: 2.07–2.18 vs. median: 2.1730, range: 2.15–2.23, *p*<0.001; Cohen’s effect size, U3 = 0.8158 (95% Confidence Intervals, CIs: 0.6316, 1.0)), the right hippocampus (median: 2.1430, range: 2.05–2.19 vs. median: 2.1760, range: 2.12–2.21, *p* = 0.004; U3 = 0.8421 (CIs: 0.5263, 1)), as well as left thalamus (median: 2.4230, range: 2.40–2.44, *p* = 0.005; U3 = 0.7895 (CIs: 0.5789, 0.9473)) in schizophrenia patients, relative to healthy individuals. Our findings provide in-vivo quantitative evidence for reduced surface complexity of hippocampus, with reduced FD indicating a less complex, less regular GM surface detected in schizophrenia.

## Introduction

**Fig 1:**
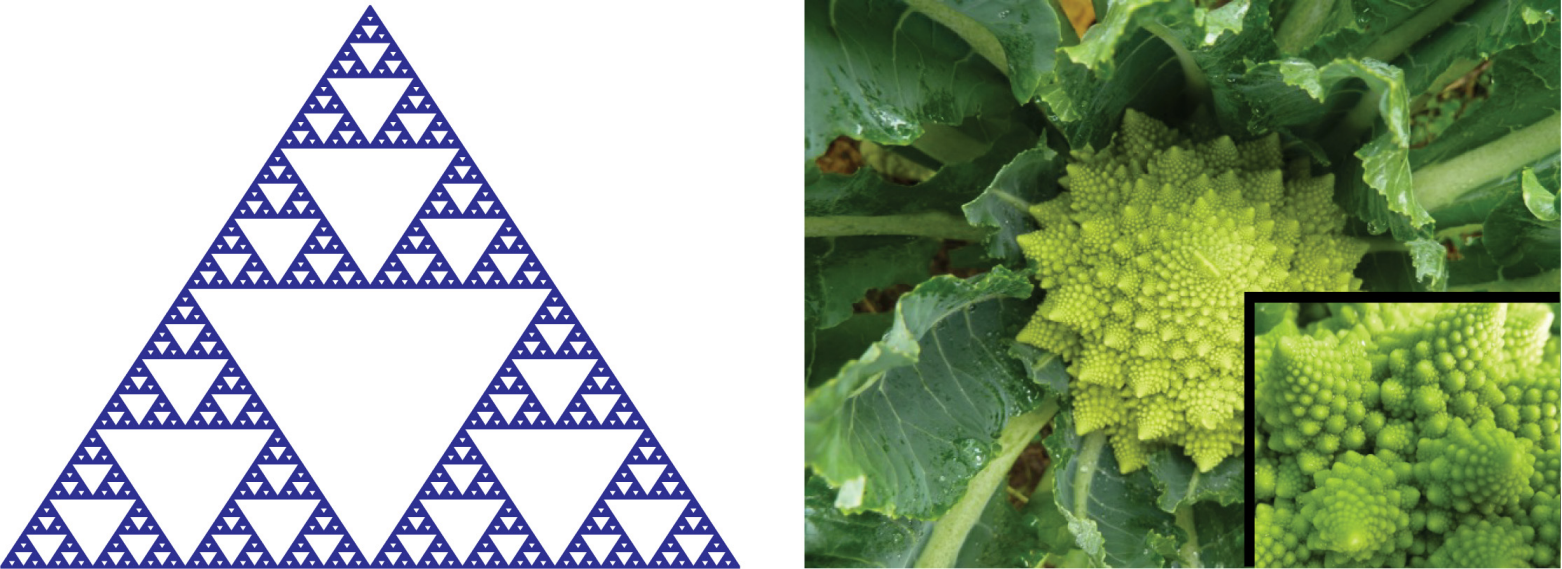
Illustration of fractal self-similarity. (a) A Sierpinski triangle is an example of a pure fractal. A small portion of the triangle looks exactly like the whole triangle. (b) Self-similarity holds across a limited range of spatial scales for a natural object such as this Romanesco Broccoli (Photos courtesy of Live Earth Farm).

The language of fractal geometry is concerned with representing the seemingly intractable aspects of form of biological objects and systems. Irregularities in biophysical signals, although typically treated as noise in most linear approaches, can be represented systematically and fruitfully using non-Euclidian geometry. In particular, the subtle variations in morphological or structural complexity of non-linear, irregular objects may be analyzed using fractal dimension (FD) measures [1, 2]. Fractal dimension characterizes sets that have a non-integral dimension. One implication of this fractional aspect of FD measure is that although one-dimensional tracings of heads of various vegetables will have an effective topological dimension of 1 (equivalent to a circle), their qualitative differences (e.g., a (smooth) head of cabbage vs. a head of romanensco broccoli in Fig 1B) will likely have discriminable fractal dimensions due to small non-topological surface variations in these vegetables. Fractal form analysis thus entails quantification of non-topological aspects of form [2]. We note that pure fractals such as the Sierpinski triangle in Fig 1 exhibit self-similarity, a scaling property that refers to invariance, or the conservation of certain statistical properties of the object’s form and structure, as the spatial scale increases (*ad infinitum* in the case of pure fractals). Intuitively, it means that when a portion of an object is magnified, the zoomed-in portion is perceived similar to the whole object (Fig 1A).

FD is a compact, “unit-less” (scale-free, following a power-law function) geometric shape descriptor of surface morphology of the object that takes into account its interior, regional properties in addition to its surface or topological features [3]. The metric yields a single numerical estimate of the overall “complexity” of an object under study, typically ranging between 2.0 and 3.0 for a 3D object. The concept of structural complexity of an object can be conceived as quantification of statistical properties of its surface structure, over successively smaller scales, when using appropriately scaled and increasingly smaller sampling or measuring instrument. Natural objects, such as the human brain, exhibit statistical fractal properties—i.e., they exhibit self-similarity only over a limited range of spatial scales [4] (See Fig 1B). Given that the shape of the brain does not follow conventional Euclidean geometry [4], for example, due to folding and convoluting, the application of standard morphological approaches to represent its shape may not be optimal, especially when aiming to capture subtle differences in gray matter structures in individuals with psychiatric disorders, in particular, schizophrenia.

The scale-free property of FD confers an important advantage over conventional volumetric approaches because it obviates the need to account for the different scaling relations that govern volumes of discrete brain structures of the human brain relative to an individual’s head size or body. For example, although it is common practice to adjust for head size in volumetric studies by covarying for the whole brain volume, this actually may be inappropriate when computing volumes of discrete brain structures, as it is the case that different structures (e.g., hippocampus) do not all scale up at the same proportions relative to increases in head size (e.g., [5, 6]). Further, it is unlikely that similar scaling factors can be assumed, even for the same structure, across individuals with and without neuropsychiatric diagnoses. We here use 3D modeling to establish that volume and FD measure outcomes are dissociable. An object with a constant volume can have distinct FD outputs (S1 Fig). Conversely, reducing the volume of an object does not increase or lower its FD measure: an object whose volume is reduced by as much as 50% yields a relatively constant FD output (S2 Fig). This means that we can characterize FD for each individual without regard for that individual’s head (or body) size or diagnostic status. Put simply, a uniform or common scale enables meaningful interpretation of measurements that arise from within as well as across typically and atypically developing individuals [7, 8].

The characteristic cortical folding or gyrification patterns—the extent of “buckling” of the initially smooth, lissencephalic cortical mantle that produces gyri and sulci—are established in utero and during early postnatal development [9]. These patterns are thought to reflect inter-neuronal organization and connectivity (e.g., [10–12]), and can be disturbed during adverse neurodevelopment [13]. Aberrant neuronal migration and synaptogenesis in schizophrenia [14–16], an absence of gliosis [17], and the presence of structural brain abnormalities in first-episode patients (e.g., [18]) have been argued to reflect aberrant neurodevelopment in this illness. Therefore, proxy measures that characterize structural surface irregularities of the brain, such as the FD surface complexity measures, might reveal information about the pathogenesis of schizophrenia that is somewhat independent of the duration or onset of illness. We first consider evidence for abnormalities in schizophrenia at two different levels of analysis.

Indeed, neuropathological findings, derived from postmortem histological analyses of the brains of patients with and without schizophrenia and conducted at the micro-level scale, include disturbances in the cytoarchitecture (the morphology and arrangement properties of cells; [19]) as well as abnormalities in the synaptic and dendritic parameters in the tissues across the brain in patients with schizophrenia. The majority of neuropathological abnormalities in the subcortical structures of schizophrenia patients have been reported in the hippocampal formation (including hippocampus proper and entorhinal cortex, EC) and, to a lesser degree, in the thalamus (e.g., [20, 21]) as well as in other subcortical structures (e.g.,[22]). In the hippocampus, examples of abnormalities include “disarranged architecture and diminution of the nerve cell-population” in the rostral entorhinal region in the parahippocampal gyrus [23], elongated, smaller pyramidal neurons in the hippocampal subfields [24], suggesting hippocampal dendritic abnormalities [19], significantly reduced synaptic density [25] and altered synaptic morphology [26], as well as reports of decreased expression of several presynaptic proteins, in particular synaptophysin (SNAP-25) and the complexins (e.g., [27]).

Although recent research has explored subtle structural differences in the shape of brain structures in schizophrenia in-vivo using Magnetic Resonance (MR) images, previous work focused primarily on characterizing cortical GM. Abnormalities in cortical folding using the gyrification index (GI, a surface analysis measure) in cortical GM in schizophrenia include both hypogyrification (e.g., [28–31]), hypergyrification (e.g., [32]), or both [33], although two very large recent studies [34, 35] found reduced gyrification in the cerebral cortex of schizophrenia patients (we note that one of these recent studies, [35], used a more sensitive, millimeter-scale measure). Fractal analyses have reported complexity reduction for the GM of the cerebral cortex [36–38] as well as for the boundary between the cortex and white matter [39], although increases in complexity (of the cerebral cortex: [40]); of the boundary between cortex and white-matter: [41]) have also been found.

In contrast to this previous work, subtle differences in the shape of subcortical GM structures in schizophrenia have not been explored systematically. The few studies that have examined differences in morphology of hippocampus in schizophrenia report mixed evidence with respect to the type of shape abnormalities found (e.g., with some studies reporting inward or outward deformations, or both in the same study) as well as the specificity of reported deformations to a discrete area within the hippocampus (e.g., anterior or posterior hippocampus). For example, early work found differences in global bending of the hippocampus [42] and exaggeration of hippocampal asymmetry pattern [43] in patients with schizophrenia relative to healthy controls. More recently, [44] and [45] reported inward deformation in the anterior hippocampus bilaterally, with [44] also reporting outward deformations, including in the inferior anterior hippocampi (bilaterally). On the other hand, [46] found outward deformation in the dorsolateral and dorsal anterior hippocampus, with inward deformations observed in the mediodorsal and the anterior and posterior regions of the ventral hippocampus. Further, [47] found surface-inward deformations in the tail of left hippocampus in patients with first-episode schizophrenia relative to healthy controls. Information on morphological properties of other subcortical structures in schizophrenia, relative to healthy controls is limited; for example, investigation by [48] found volume compression in the lateral and dorsal and the middle body of the thalamus (bilaterally) in first-episode patients with schizophrenia. While these studies point to the existence of morphological abnormalities (e.g., in particular, of hippocampal morphology), they do not provide an unequivocal consensus with regard to the specificity and directionality of abnormalities that are detectable in-vivo, for the structures under examination.

It is possible that differences in subcortical morphology vary across samples because subtle structural aspects of hippocampus may be highly variable across individuals with schizophrenia. Further, previous work on shape analyses reviewed above actually requires smoothing the structure in order to remove potential noise from the surface (see [47, 49]) because only smooth structures could be utilized as input by currently used shape analysis algorithms. If one postulates that irregularities in the structure exist at the surface, as we do here, a smoothing step would effectively treat any irregularities as noise. In contrast, FD approaches are able to model the structure without requiring the input to be smooth. One may also argue that it may be challenging to apply currently used gyrification measures to study the gyral pattern of subcortical structures because these measures estimate complexity of the cortical mantle (the frequency of the cortical folds, gyri and sulci) at the relatively coarse centimeter-level scale (but see Ronan and colleagues work [35] for finer-resolution, millimeter-level scale approach). To overcome these potential caveats, we estimated a single, quantitative, numerical index, FD—for subcortical structures in individuals with and without schizophrenia.

### Current study

Here we use fractal analysis in order to systematically explore and quantify evidence for subtle differences in the shape of seven subcortical GM structures (hippocampus, caudate, putamen, pallidum, nucleus accumbens, thalamus, amygdala—bilaterally, as well as the brainstem) in patients with schizophrenia compared with healthy controls. In particular, the hippocampus is a highly convoluted and evolutionarily older structure compared to the neocortex, contributing to encoding, consolidation, and retrieval of declarative memories [50], the representation of the temporal context (e.g., [51]) and working memory (e.g., [52]). Because of the extensive involvement of each of these subcortical structures in normal sensory processing and the substantial interaction of these regions with each other as well as with the cerebral cortex (please see S1 Text for details), our goal was to investigate whether potential subtle alterations are detectable in-vivo for some or all of these structures at the group-level within the same sample of schizophrenia patients relative to healthy controls.

In the context of solid objects, FD characterizes macroscopic surface structural properties of matter and represents an “index of the morphometric variability and complexity of an object” [3]. This means that information is provided about variability of the surface structure of the object across different scales as well as about variability (i.e., via figure/ground relation) of white matter proximal to that structure. As an example, a surface that is completely lacking in texture or complexity would be smooth and liccencephalic, whereas a surface that has some texture would be characterized by protrusions or “bumps” (convexities) and indentations (concavities). The “bumps” could derive from aggregations of neuronal nuclei, dendrites, as well as axonal projections to other structures. The indentations would suggest impingement of the surrounding matter into the structure. As noted in [53], however, either polymicrogyria (too many small gyri or protrusions) or pachygyri (too few large gyri) are indicative of abnormalities relative to a typically developing, healthy convoluted brain [53, 54]. Deviations in the complexity of folding—either increases or decreases—would suggest an abnormality.

We sample the surface of each structure to detect the probability of protrusions of a given size using gradually varying resolutions (increment sizes). The likelihood of finding a protrusion of a given size, relative to a range of sizes, follows a power-law function. In essence, this analysis uses probabilistic estimation of the number of counts (per size of the protrusions), across gradually increasing (size-wise) increments. This technique captures the statistical complexity of each structure (FD; expressed as the slope of the best-fit line on the double logarithmic axes). The deviation from non-linearity in the increases (or decreases) of the sizes of observed protrusions is quantified by the goodness-of-fit statistic (please see Methods for the details of the analytic approach). We hypothesized to find aberrant structural surface complexity in subcortical GM in the schizophrenia group and expected that the index of morphometric variability (FD) will deviate relative to healthy controls, in particular with regard to hippocampi. In this exploratory work, the two predictions consistent with our bi-directional hypothesis is that relative to FD complexity outcome in healthy controls, in schizophrenia patients, either (*i*) complexity is reduced, or (*ii*) complexity is increased. The competing hypothesis is that FD values would be similar between patients and healthy controls.

## Materials and Methods

### Participants

Participants included 19 individuals meeting Diagnostic and Statistic Manual of Mental Disorder (DSM-IV) criteria for schizophrenia (SCZ) and 19 healthy control volunteers (HC). Diagnoses were based on the Structured Clinical Interview for DSM-IV (SCID) [55] as well as available clinical information. Individuals in our SCZ sample were patients with chronic schizophrenia (mean duration of illness 15±9.8 years) who were on antipsychotic medication; this sample’s duration of illness and medication intake is comparable to individuals from our other research studies in schizophrenia [56]. Patients were recruited from inpatient and outpatient facilities affiliated with the Nathan Kline Institute, while HCs were recruited from the Nathan Kline Institute’s Volunteer Recruitment Pool. Exclusion criteria for healthy volunteers was a history of Axis-I psychiatric disorders based on the SCID interview. Healthy volunteers were also required to have no current Axis-I psychopathology, based on SCID. Potential patient and healthy control participants were excluded if they had any neurological disorders or had contraindications to the MRI environment (e.g., metal implants or claustrophobia), or IQ less than 70 [57]. Despite the requirement to have normal IQ (>70), patients with schizophrenia had a significantly lower IQ (94 ±7.8) relative to healthy controls (103 ±11) (*p*<0.0062). Additional exclusion criteria for both patients and healthy volunteers were substance abuse or alcohol dependence in the last 6 months, or abuse within the last month. Participants were matched on age (SCZ: 39.59±9.78, HC: 35.79±8.51, *t*(36) = 1.28, *p* = 0.21), and did not significantly differ in gender distribution (SCZ: 17 males, 2 females; HC: 13 males, 6 females; *Χ*^*2*^(1, N = 38) = 2.53, *p* = 0.11). This study was approved by the Institutional Review Board at the Nathan Kline Institute for Psychiatric Research. Prior to consent procedures, the capacity to provide informed consent was determined for participants with schizophrenia by a licensed psychiatrist with extensive experience working with patients with schizophrenia. After full explanation of study procedures, all participants provided informed written consent prior to beginning participation in the study. The study was carried out in strict accordance with the Code of Ethics of the World Medical Association (Declaration of Helsinki).

### MR acquisition

High-resolution T1-weighed structural whole-brain Magnetic Resonance Images (MRI) were acquired on a 3T Siemens scanner using an 3D magnetization prepared rapid gradient echo (MPRAGE) pulse sequence using the following parameters: image matrix 256 x 256, 1x1 mm^2^ in-plane resolution, 192 saggital slices of slice thickness 1mm, no gap, Repetition Time (TR) and Echo Time (TE), TR/TE = 2500/3.5ms, and inversion time (TI) = 1200 ms, and flip angle = 8°.

### Preprocessing and Segmentation procedure

It is important to minimize potential differences in pose variance of images entering FD calculations as these have been noted to affect FD estimates [58]. Therefore, in order to reduce variable effects of different and non-orthogonal orientations of images (cf. [59]), T1-weighted structural images were registered to standard stereotaxic space, as follows. T1-weighted structural images were initially processed with SPM8 (http://www.fil.ion.ucl.ac.uk/spm) using VBM8 toolbox (http://dbm.neuro.uni-jena.de/vbm8) running MATLAB 8.3 (R2014a) (MathWorks, Natick, MA, USA). We used fully automated procedures in VBM8 for bias field correction and skull-stripping in order to remove non-brain tissue from T1 images. In VBM8, T1-weighted structural images were bias field corrected first and then were skull-stripped, following alignment from native space to a Montreal Neurological Institute standard space (MNI-152 template) [60]. VBM8 DARTEL’s registers T1 images to MNI space using tissue probability maps (ICBM) with resolution of 1.5×1.5×1.5 mm^3^ (hence, our T1-weighted images were therefore resampled to 1.5×1.5×1.5 mm^3^ (new image matrix: 121 x 145 x 121)).

VBM8 has several pre-processing pipeline options, including non-linear (DARTEL) and linear (non-DARTEL) registration of T1 images to MNI space; we used VBM8 DARTEL’s ‘modulated normalized’ option as it involves not only normalization of the images but also their subsequent "modulation". As a result of nonlinear spatial normalization of T1 images to MNI space, the volumes of certain brain regions may change. The “modulation” step compensates for the effects of spatial normalization (i.e., produced by affine transformation (global scaling) and non-linear warping (local volume change)). Specifically, this further processing step attempted to preserve the volume of a particular tissue within a voxel. This consisted of “multiplying (modulating) voxel values in the segmented images by the Jacobian determinants derived from the spatial standardization step” [61].

Following brain extraction and spatial normalization steps, subcortical structures were segmented (from image output in MNI space, generated by the VBM8) using fully automated FMRIB’s Integrated Registration and Segmentation Tool (FIRST) tool in the FMRIB Software Library (FSL) (version 4.1, University of Oxford, England; http://www.fmrib.ox.ac.uk/fsl) [62].

The reason why we used SPM’s VBM8 for skull-stripping instead of using FSL FIRST itself for this procedure was due to a relatively poor performance of FSL FIRST on skull-stripping. FSL FIRST uses Brain Extraction Tool (BET) [63] to remove non-brain tissue. However, BET seems to suffer from a thresholding issue (a larger threshold would cause removal of brain tissue, but a smaller threshold would lead to incomplete removal of non-brain tissues); BET also induced some distortions in the skull-stripped image output.

FIRST uses a training set comprised of manually traced subcortical structures from 336 demographically diverse subjects (age range: 9 to 87), including healthy, typically developing and typically aging individuals as well as pathological cases, including patients with schizophrenia and Alzheimer’s disease [62]. Briefly, this subcortical brain segmentation tool is based on a Bayesian Active Appearance Model that extends previous Active Shape Model (ASM) (e.g., [64]) and the Active Appearance Model (AAM) [65], incorporating intensity information as well as the geometrical shape (an important characteristic considering increased susceptibility, to imaging artifacts, by subcortical structures). Segmentation in FIRST exploits the relationship between intensity and shape within a probabilistic framework; non-informative priors are used to avoid bias (for additional details on the segmentation approach, please see [62]). FIRST segmentation was conducted using all standardized procedures, using the run_first_all routine with a “-b” option to use a skull-stripped image as input (for a detailed description, see [62]). Boundary correction subroutine in run_first_all was used to classify voxels as belonging to the structure (*Z*>3.00, *p*<0.001). This segmentation process yielded volumetric image outputs in MNI space [62] for each subcortical structure as well as the brainstem, which were then discretized as binary mask images for input to our FD algorithm. The fslstats routine was used to obtain the volume of the subcortical gray matter structures.

### Fractal analysis

We estimated fractal dimensions of subcortical structures based on the box-counting method, which has been recently validated for optimization and reproducibility by [59], including analysis of subcortical structures in healthy individuals. Box-counting is (*i*) simple, accurate, and robust, (*ii*) can evaluate objects with and without self-similarity, and is (*iii*) particularly suitable to measure dimensions of sets of points or volume elements [58, 66]. Here, a set denotes a three dimensional (3D) subcortical gray matter (GM) structure formed by voxels. Measuring the fractal dimension of a set begins with fully covering it with 3D grids of cubes of regular, fixed sides of length *r*, for a specified range of values of *r* (*described below*). We first detect a voxel on the structure in the x, y, or z direction, and then align the 3D grid to this voxel. Thus, grid position is aligned with the subcortical structure, not the edge of the image. For each differently sized 3D grid, the number of cubes that *contain part of the set* is counted (i.e., *estimated* probabilistically) and recorded. Thus, distinct sizes of cubes of different 3D grids represent the different scales or resolutions applied to the set. Intuitively, “dimension” is a scaling exponent of a given *set* with its size (across different scales or cube sizes): *set* ∝ *size*^*Dimension*^. This relation states that the frequency of finding a portion (e.g., a protrusion) of the object of a given size is proportional to the set’s size. (We note that in the context of the Information Dimension, as explained below in detail, the general relation between cube size *r* and dimension is inversely proportional, and would be given by: *I* ∝ *r*^−*D*1^).

Briefly, we note that determining the initial range of box sizes is important because inappropriate values have been shown to lead to biases in fractal estimation (e.g., for example, in the case of dispersed or noisy images [58]). In particular, the minimum and maximum box sizes should not be very small or very large. Specifically, in the present work, the minimum box size (*Min r*) is 2 voxels (as suggested in [3], while the maximum box size (*Max r*) is 30 voxels, equal to the 25% of the shortest side of the image for each subcortical structure [58], yielding 3D grids of side length in the range of *r* = 2 … 30 voxels. Our algorithm routine starts with the minimum box size and iteratively proceeds to increasingly larger box sizes, up to the maximum box value, in increments of 1 voxel.

In the current work we use a probabilistic method for computing FD because it has been shown to have a higher inter-class correlation [59]. The information dimension, *D*_1_, uses information theoretic entropy to represent inhomogeneities [67], that is, it distinguishes between a box that contains many points of the set or only a few. The uncertainty in a collection of possible states *I* with the corresponding probability distribution *P*(*i*) is given by its entropy [68]. In Information Theory, entropy (formally the Shannon entropy) is a measure of uncertainty [68]. Entropy characterizes the amount of information in the data. We note that the term “information” denotes the statistical aspects of data with regard to the amount of choice or freedom of possible outcomes given an information source and the uncertainty about the choice actually made; the smaller the probability of an event, the more information it provides when it occurs.

The total number of voxels of the fractal object can be expressed as *N*_*all*_. Let us also define *N*_*i*_(*r*) as the number of voxels which are covered by the *i*-th box in a 3D grid of regular sides of length *r*, in the range *r* = 2 to 30 voxels (in increments of 1). *P*_*i*_ is the probability of finding a voxel of the fractal object falling into (or intersecting with) the *i*-th box in a 3D grid of side *r* and can be expressed as:

$$p_{i}=N_{i}(r)/\;N_{all}.$$ (1)

We compute the entropy *I*_i_(*r*) for the *i*-th box of size *r* (*r* = 2 to 30 voxels; in increments of 1):

$$I_{i}(r)=-p_{i}\;\text{log}(p_{i}).$$ (2)

And, *I*(*r*) for each box of size *r* can explicitly be written as:

$$I(r)=-\sum_{i=1}^{N(r)}p_{i}\;\text{log}(p_{i}).$$ (3)

Thus, *I*(*r*) provides the “information (in bits), needed to specify a point on the set to an accuracy *r*” [69]. The information dimension *D*_1_, is defined as:

$$D_{1}=\underset{r\to 0}{\text{lim}}\;\text{log}(I(r)/\text{log}(1/r).$$ (4)

In computing FD of solid fractals, lim *r* → 0 is not attainable [69] because the box size *r* cannot be arbitrarily small (i.e., the finite resolution restricts the lower bound of the box-size range). Therefore, FD computations are performed over a restricted range of box values, *with minimum r = 2*, as noted above. In the final step, the Fractal Dimension (FD) measure is obtained by fitting a linear regression (least squares) on the scatter plot of log(1/*r*) versus log(*I*(*r*)).

**Fig 2:**
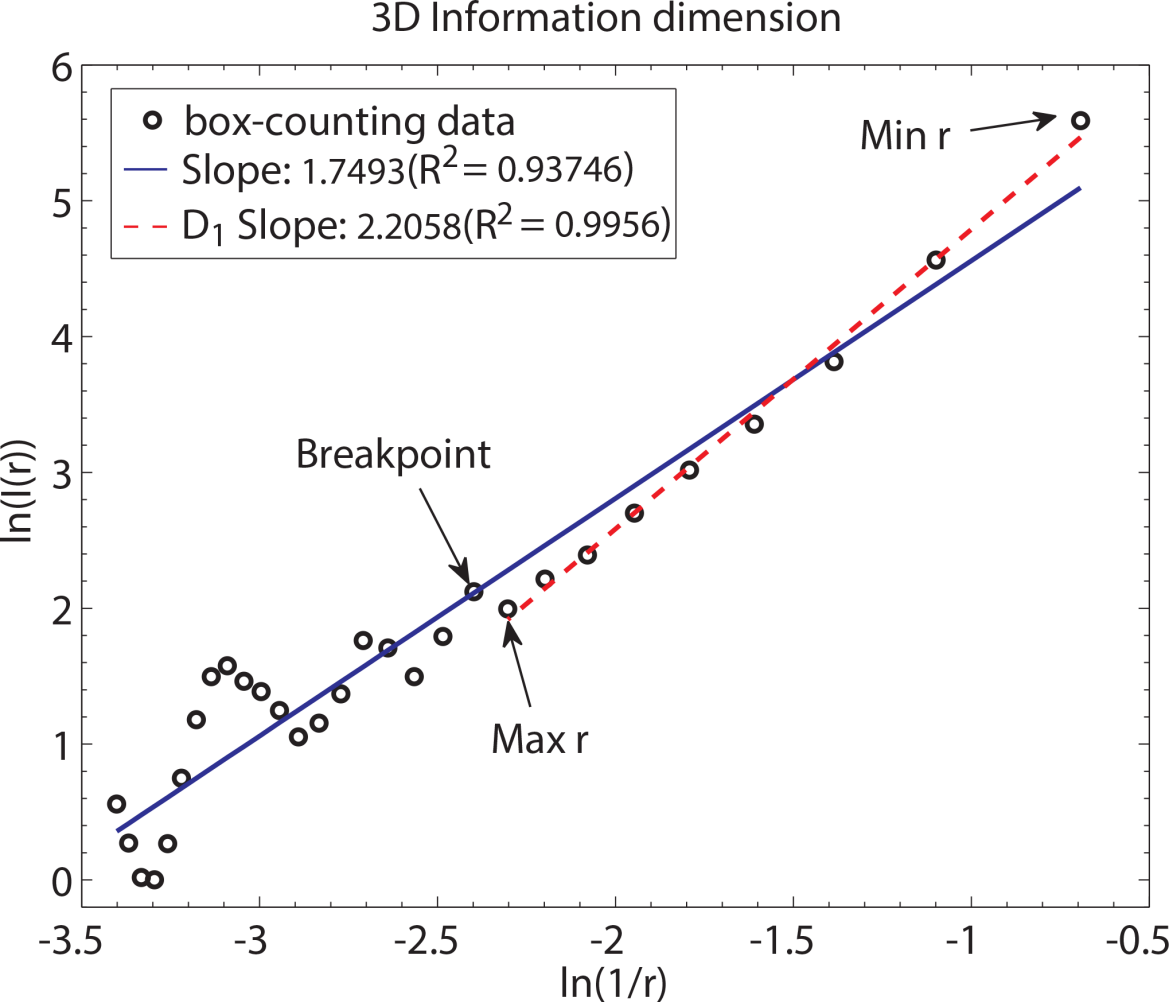
Computing fractal dimension using 3D information measure. Information dimension, *D*_1_, measure. In the scatterplot of log(1/*r*) versus log(*I*(*r*)), *r* is box size and *I*(*r*) is the information theoretic entropy for the box size *r*. *Note*. For information measure, the initial, pre-determined range of box sizes is *r* = 2… 30 voxels (in increments of 1 voxel). Data shown are for left hippocampus from one healthy control participant. Linear regression analysis is performed iteratively. Blue line indicates the linear fit over the entire range of *r*. Red dotted line indicates the final fit (*R*^2^); the slope of this line corresponds to the fractal dimension, *D*_1_. Breakpoint separates non-linear data points from the data used in the final regression analysis. ln denotes natural log. *Min r* is the new smallest box size and *Max r* is the new largest box size.

The log-log plots are initially plotted using the entire range of box sizes that have been determined *a priori*, and the initial FD estimate is computed as the slope of the log-log regression line. Points that deviate from the line of regression fit in the log-log plot appear as a non-linear distortion in Fig 2. Because such points do not provide further complexity information about an object, regression fits are repeated, by systematically excluding these points, until a straight line that includes most data points can be fitted [58]; the final fit is chosen so that it produces the largest *R*^2^.

We have found that data points comprising the non-linear portion correspond to the larger box sizes (corresponding to smaller log(1/*r*) values on the x-axis in Fig 2). Specifically, *I*(r) estimates at large box sizes show oscillatory behavior, in that *I*(r) estimates do not scale linearly as a function of increased box size *r*. Although our finding of this pattern is consistent with earlier observations [3], the exact reason for the oscillatory behavior of *I*(r) estimates at large *r* has not been thoroughly investigated, and presents an area of investigation for future work.

Previous investigations [3] suggest that for small subcortical structures, 3D grids with the smallest box sizes are most appropriate. For the 39 participants in the study, no box sizes larger than 15% of the smallest side of the image (corresponding to *r* = 18 voxels), intersected with more than 85% of the image. Therefore, we suggest that, from a computational efficiency standpoint, this size suffices as the upper bound for the largest box size, for small subcortical structures.

The point that distinguishes the linear and non-linear parts of the data is the breakpoint (indicated by the arrow in Fig 2). The exact position of the breakpoint in the log-log plot is different for each subcortical structure. The data points utilized for the final linear regression fit include the revised upper bound (i.e., largest box size, *Max r*); in some cases, the lower bound (i.e., the smallest box size, *Min r*) may also be revised in order to obtain the largest *R*^2^; see Fig 2. The slope of this final regression corresponds to the FD estimate (Fig 2). Although all structures were highly linear during linear regression analyses, left and right hippocampus were more stable. The coefficient of determination (*R*^2^) for the linear regression model fits to the data were as follows: left and right hippocampus >0.99, left and right thalamus >0.985, and all other structures >0.985. There were no systematic differences between the patient and control group in the breakpoint above which the data were judged linear, for all subcortical structures (all *p*>0.05).

The algorithm was implemented using routines written in MATLAB 8.3 (R2014a). In summary, the routine directly reads FIRST subcortical structure files, performs probabilistic counting for each 3D grid with box length *r*, computes slopes in a recursive manner, and determines the final slope value (i.e., the FD estimate) based on the straight portion of the line for each subcortical structure. We computed information dimension (*D*_1_) for seven subcortical structures (thalamus, caudate, putamen, pallidum, hippocampus, amygdala), bilaterally, as well as the brainstem, for patients with schizophrenia and healthy controls.

### Validation of the algorithm

**Fig 3:**
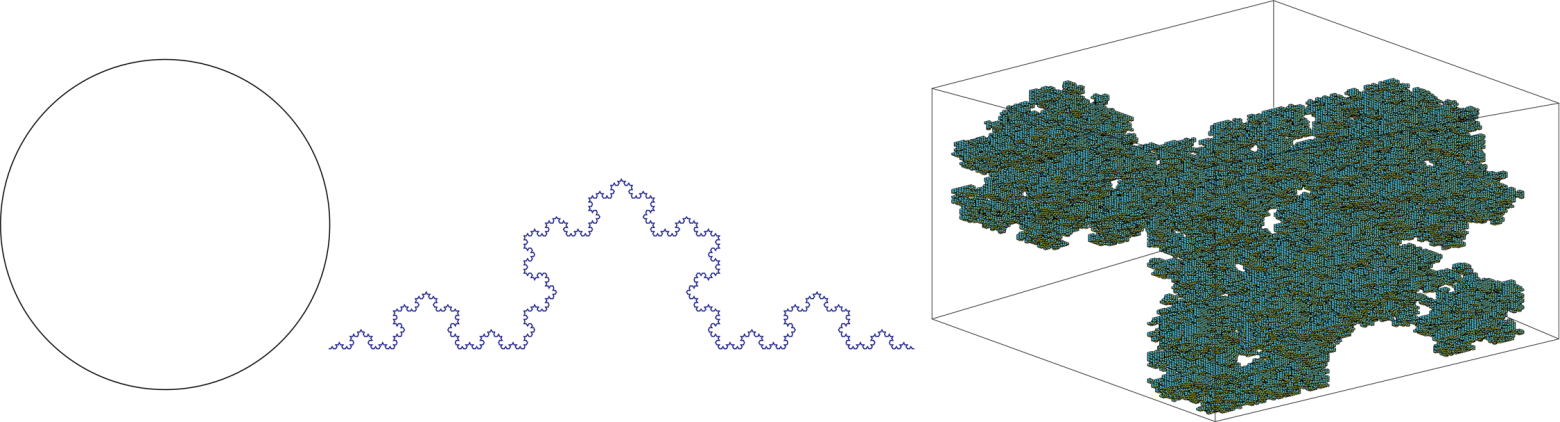
Simulated phantoms used in algorithm validation, with theoretical fractal dimensions ranging between 1 and 3. (a) Circle: radius = 8, image size: 120 x 120, line width is 1 pixel (theoretical FD = 1). (b) Fourth-iteration Koch, image size: 283 x 84, line width is 1 pixel (theoretical FD = 1.2619). (c) 3D random Cantor set with *p* = 0.7, image size: 128 x 128 x 128, Voxels set to 1 (theoretical FD = 2.485).

We validated our algorithm by evaluating three objects with known theoretical fractal dimensions: Circle, fourth-iteration Koch curve, and 3D random Cantor set (Fig 3). We constructed phantoms using MATLAB using the following parameters. The circle had radius 8, with an image size 120 x 120; pixels of the circle were set to 1. The fourth-iteration (n = 4) Koch curve had image size 283 x 84; pixels of the Koch were set to 1. The 3D random Cantor set had image size 128 x 128 x 128 with *p* = 0.7; voxels of the Cantor were set to 1. Empirical information FD computation produced close estimates relative to the theoretical fractal dimensions of the phantoms (Table 1). The values of *D*_1_ (0.9955 for the circle, 1.2699 for the fourth-iteration Koch and 2.4361 for the 3D random Cantor set) of phantoms are close to their theoretical dimensions (Circle: ~1.0, fourth-iteration Koch, log(4)/log(3): ~1.2619, and 3D random Cantor set, 3 + log(*p*)/log(2): ~2.485). We did not expect a perfect match to the theoretical value because the image is only an approximation of the physical form of the object.

**Table 1:** Fractal dimension values and box size range of phantoms.

	Expected FD	Computed FD (box range)
Circle	1	0.9955 (2–16)
4th-iteration Koch	1.2619	1.2699 (2–18)
3D random Cantor set	2.485	2.4361 (2–9)

Fractal algorithm evaluation. Theoretical and empirical values for information fractal dimension measure. Values in parentheses indicate the final box range used in the FD estimation. FD: Fractal Dimension.

### Statistical analysis

Normality of distributions was assessed using the Shapiro-Wilk test; non-parametric tests were used for group analyses because data did not satisfy the requirements for a normal distribution. Group statistical differences in local *D*_1_ measures were assessed using a Mann-Whitney *U* test (two-tailed). False Discovery Rate (FDR) procedure was used to correct for multiple comparisons (FDR q<0.006 [70, 71]) across the fifteen output values (seven left and right GM structures, and the brainstem). Significant between-group results were examined post-hoc using linear regression, testing for interactions between age and sex with group diagnostic status. The models included FD as the dependent variable and group, age, and group*age (or sex and group*sex) as predictor terms: Ŷ = β_0_+ β_1_(group)+ β_2_(age)+ β_3_(group*age) or Ŷ = β_0_+ β_1_(group)+ β_2_(sex)+ β_3_(group*sex). Correlation coefficients were computed to test the association between complexity and volume (Spearman's *rho*). (The level of significance was set to *p*<0.05). All statistical analyses were performed using SPSS (IBM SPSS 23.0, Armonk, NY: IBM Corp.), except for Cohen’s effect size (U3, a non-parametric measure of effect size that makes no assumptions about underlying data distributions [72]) which was computed using Effect Size Toolbox (version 1.4) [73] running Statistics Toolbox in MATLAB.

## Results

**Fig 4:**
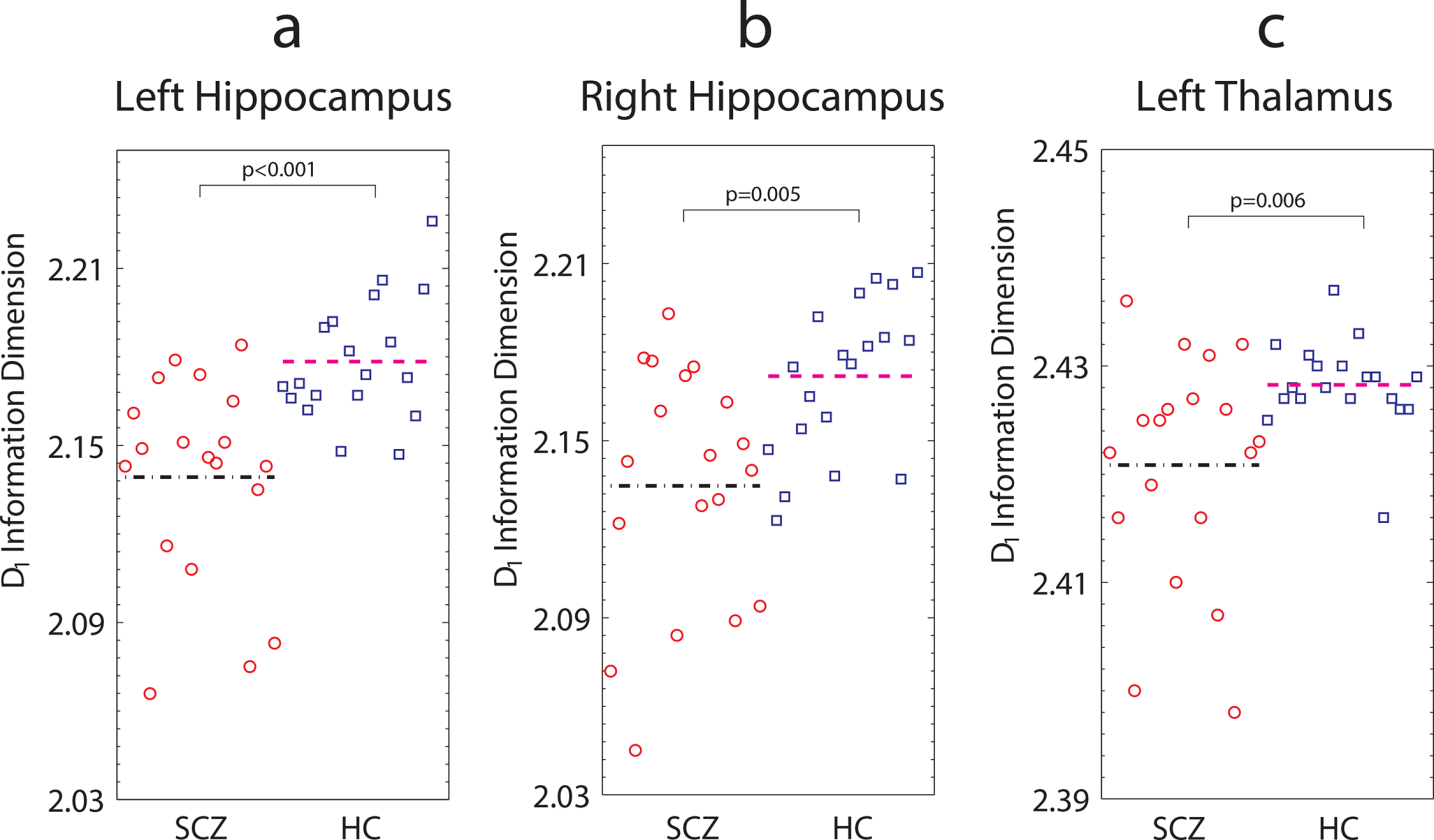
Differences in fractal dimension for left hippocampus, right hippocampus and left thalamus between schizophrenia patients and healthy controls. Each data point represents *D*_1_ information dimension value for each participant for (a) Left hippocampus, (b) Right hippocampus, and (c) Left thalamus. The black dash-dot line and the magenta dash-dash line denote median fractal dimension values, for schizophrenia patients and healthy control groups, respectively. Significantly lower FD values were found for schizophrenia patients relative to healthy controls (Mann-Whitney *U* test, *p*< 0.05; FDR correction). *Note*. SCZ: patients with schizophrenia; HC: healthy controls.

Table 2 presents FD values for all GM subcortical structures, for patients (SCZ) and healthy controls (HC). Because *D*_1_ is the slope in the log-log plot (ln(*I*/r)/ln(1/r); see Methods for details), it reflects a relation between the estimated (observed) sizes of protrusions at different scales (box sizes). We detected significantly lower information dimension, *D*_1_, values in the left and right hippocampus and left thalamus in patients with schizophrenia compared with healthy controls, as shown in Fig 4.

**Table 2:** Fractal dimension values for subcortical structures.

		Left						Right				
Structure		median	min	max	*U*	p		median	min	max	*U*	p
Thalamus	SCZ	2.423	2.398	2.436	86.5	0.005*	SCZ	2.402	2.364	2.421	132.5	0.163
	HC	2.428	2.416	2.437			HC	2.405	2.396	2.418		
Caudate	SCZ	2.2	2.136	2.261	134.5	0.181	SCZ	2.216	2.034	2.266	134.5	0.181
	HC	2.210	2.177	2.232			HC	2.190	2.107	2.257		
Putamen	SCZ	2.37	2.347	2.42	173.5	0.840	SCZ	2.269	2.245	2.307	135.5	0.191
	HC	2.375	2.32	2.43			HC	2.273	2.237	2.311		
Pallidum	SCZ	2.146	2.061	2.373	127	0.123	SCZ	2.16	2.067	2.215	110	0.040
	HC	2.121	2.078	2.200			HC	2.117	1.927	2.184		
Hippocampus	SCZ	2.146	2.066	2.184	56	0.000*	SCZ	2.143	2.045	2.193	84.5	0.004*
	HC	2.173	2.15	2.23			HC	2.176	2.123	2.207		
Amygdala	SCZ	2.347	2.28	2.404	141.5	0.258	SCZ	2.345	2.204	2.446	149.5	0.370
	HC	2.339	2.265	2.376			HC	2.356	2.278	2.427		
NucleusAccumbens	SCZ	1.976	1.851	3.012	139.5	0.234	SCZ	2.093	1.785	2.402	133	0.172
	HC	2.000	1.827	2.648			HC	2.156	2.041	2.349		
Brainstem	SCZ	2.47	2.455	2.678	129.5	0.138						
	HC	2.467	2.451	2.508								

Fractal dimension (*D*_1_) structural complexity values (median and range: min and max) are shown for the seven subcortical GM structures (for Left and Right hemispheres), and the brainstem, for schizophrenia patients (SCZ) and healthy controls (HC). *Note*.

* indicates that Mann-Whitney *U* test results survived FDR correction.

We consider these results as follows. FD reduction indicates a shallower slope, reflecting fewer estimated *I*(r) counts across box-size increments, (r), for the patient group. Specifically, a Mann-Whitney test indicated significant differences in FD between patients and controls in the left hippocampus (FD was lower in SCZ patients (median: 2.1460, range: 2.07–2.18) relative to HC (median: 2.1730, range: 2.15–2.23), *U* = 56, *p*<0.001), the right hippocampus (FD was lower in SCZ patients (median: 2.1430, range: 2.05–2.19) relative to HC (median: 2.1760, range: 2.12–2.21), *U* = 84.5, *p* = 0.004), and the left thalamus (FD was lower in SCZ patients (median: 2.4230, range: 2.40–2.44) relative to HC (median: 2.4280, range: 2.42–2.44), *U* = 86.5, *p* = 0.005). (We also found a marginally significant between-group difference in the right pallidum (SCZ median: 2.16, range: 2.067–2.215 vs HC median: 2.117, range: 1.927–2.184, *U* = 110, *p* = 0.040), however, we note that the comparison did not survive significance testing after removing an outlier from the HC group).

Cohen’s U3 effect size measures the proportion of data points in the lower group (here, the patient group) that are lower relative to the median of the higher group [73]. The effect size for the left hippocampus was: U3 = 0.8158 (95% Confidence Intervals, CIs: 0.6316, 1.0), for the right hippocampus, U3 = 0.8421 (CIs: 0.5263, 1), and for the left thalamus, U3 = 0.7895 (CIs: 0.5789, 0.9473). Here we a find a large effect, meaning that the overlap is minimal between patients and controls for all three significant structures [72, 73].

**Fig 5:**
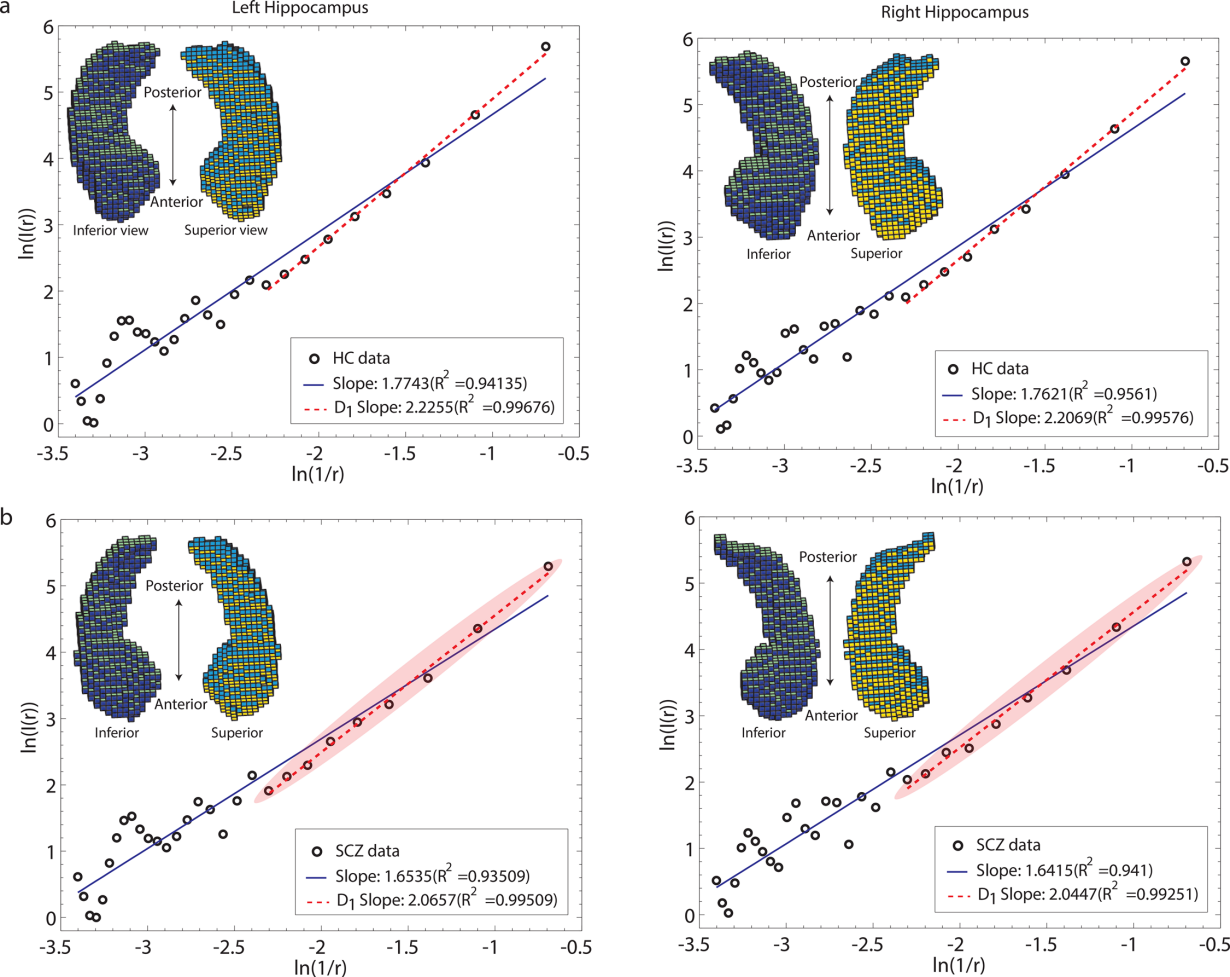
Illustration of subtle surface non-linearities in schizophrenia as captured by the FD measure, using individual participants’ data for left and right hippocampus. (a) Representative healthy control, and (b) Patient with schizophrenia. Left panel shows left hippocampus, right panel shows right hippocampus. In (b), shadow highlight indicates data points used in the final fit. Subtle deviations from linearity are seen in (b), which shows data points whose *I*(r) counts deviate relative to the line of best fit. In the left panel, an example of this can be observed at a point with x and y coordinates [-1.946, 2.652] (fourth from the bottom of shaded area) and at [-1.386, 3.608] (third from the top), and in the right panel, at [-2.079, 2.445] (third from the bottom) (please see main text for detailed explanation). Insets show reconstructions of left and right hippocampi for these participants; 1 cube represents 1 voxel (1.5 x 1.5 x 1.5 mm).

Further, the final regression fits, *R*^2^, although highly linear, showed non-linearities in patients with schizophrenia for left and right hippocampus. The fits were, for the left hippocampus: SCZ: 0.9952±0.00007, HC: 0.9958±0.0008, *p* = 0.02, and for the right hippocampus: SCZ: 0.9950±0.0011, HC: 0.9958±0.001, *p* = 0.02 (no between-group difference was found in the left thalamus, *p*>0.05). Reduction in *R*^2^ means that across different boxes of size r, *I*(r) estimates (y-axis in Fig 5) do not increase linearly (monotonically) with increasing box-size increment in the patient group relative to healthy controls, who show more uniform increases of *I*(r) estimates across increments, a subtle trend reflected in significant (*p* = 0.02) between-group differences in *R*^2^.

We illustrate this effect of non-monotonicity or irregularity with individual data plots and renderings in Fig 5. Fig 5A shows a representative healthy control participant and Fig 5B illustrates a participant with schizophrenia (left and right hippocampi are presented in the left and right panels, respectively). Data points comprising the line of best fit are highlighted with shadow in the case of schizophrenia participant. Notice that in Fig 5B, there are points that deviate from the final line of best fit. In the left panel, considering a fourth point from the bottom (within the highlighted area: x and y coordinates [-1.946, 2.652], its *I*(r) count is larger than expected, whereas *I*(r) count for [-1.386, 3.608] (third from the top) is smaller than expected. In the right panel, *I*(r) count for [-2.079, 2.445] (a third point from the bottom within the highlighted area), is larger than expected.

We then examined whether there were any systematic subgroups of individuals for whom FD is particularly abnormal (reduced) in Fig 4. (For the two main regression models, residuals of β predictor coefficients were approximately normally distributed). Sex did not interact with diagnostic group (all *p*>0.05). For the left hippocampus, no age by group interaction was detected (all *p*>0.05). For the right hippocampus, age interacted with group (β = -0.003, *t*(34) = -2.525, *p* = 0.016). Lower FD values were associated with increased age in individuals with schizophrenia: β = -0.002, *t*(17) = -2.2578 (*p* = 0.02), whereas no association between FD and age was found for healthy controls: β = 0.001, *t*(17) = .948 (*p* = 0.356). For the left thalamus, age also interacted with diagnostic group status: β = -0.001, *t*(34) = -2.277 (*p* = 0.029). Older individuals with schizophrenia were found to have lower FD values β = -0.001, *t*(17) = -2.989 (*p* = 0.008); no association between FD and age was found for healthy controls: β = -0.00005, *t*(17) = -.496 (*p* = 0.626).

For both groups, *D*_1_ and volume measures for left hippocampus, right hippocampus, and left thalamus were significantly positively correlated (*p*<0.05) (S1 Results).

Because our goal was to study FD outcome measures for all subcortical GM structures, we also looked to see if there was a systematic relationship between the size of the structure (its volume) and variability of FD output (standard deviation). This could reveal part of the reason for not finding between-group differences in the rest of the GM structures. Small subcortical structures (e.g., nucleus accumbens) were accompanied by increasing variability of FD. We find that in general, the smaller the volume of the object, the greater the standard deviation of FD (S3 Fig). We find a similar trend for both groups (all *p*<0.05). We discuss the potential limitation of FD analysis with regard to very small objects in the next section.

## Discussion

We used fractal analysis to quantify millimeter-scale morphological complexity of seven subcortical structures and the brainstem in patients with schizophrenia relative to healthy individuals. Previous work has documented reductions in GM complexity of the cerebral cortex, as well as of the subcortical and white-matter boundary in schizophrenia [36–39]. Further, morphometric studies of subcortical GM structures have identified abnormalities in the shape of the hippocampus (e.g., [42, 43, 45]) and in the thalamus [48] in schizophrenia patients. However, the directionality of shape abnormalities detected by the previous investigations—with respect to the type of deformations (inward or outward)—has been equivocal (e.g., [44]). Here we looked for evidence of subtle structural abnormalities in subcortical GM in-vivo using fractal geometry approach, computing a single FD measure for each of these structures for patients and controls. We report a novel finding, a significant reduction in the FD of bilateral hippocampi as well as left thalamus in our sample of patients with chronic schizophrenia relative to healthy controls, providing in-vivo quantitative evidence for subtle differences in morphology of these structures.

Lower complexity of subcortical GM in patients with schizophrenia indicates reduced presence of millimeter-level “protrusions” or undulations at the surface across the range of all sizes (resolutions) considered, therefore requiring fewer fine-, medium-, and coarse-grained boxes that were needed to characterize higher surface complexity of normal controls (a shallower slope can be observed for a representative SCZ patient in Fig 5B). As noted in Yotter in the context of cortical GM [37], cortical regions with greater complexity are more regular in appearance, similar to a sine wave [37]. We also found that box-count estimates did not scale as linearly with increased box-size increments in the patient group as they did in HC, suggesting an atypical or irregular distribution of differently sized millimeter-level gyri, at a given resolution considered, across the surface. We next consider our findings within the context of the aberrant neurodevelopmental hypothesis of schizophrenia.

In particular, post-mortem studies of patients with schizophrenia have documented changes in the populations of interstitial white matter neurons (IWMN), including altered density of IWMN underlying dlPFC (e.g., [74]) and altered distribution underlying the parahippocampal gyrus [75]. The IWMN neurons are traces of the subplate, a transient embryonic structure that guides neuronal migration and participates in establishing initial cortico-cortical and cortico-subcortical synaptic connections. Critically, the anomaly of the subplate in schizophrenia has been argued to contribute, in part, to aberrant short- and longer-range connectivity across the brain, and aberrant gyrification—assuming that gyrification arises in part due to inter-neuron connections [9] (although the exact conditions under which gyri and sulci form are not fully understood). This is relevant because an aberrant process, whether limited to early life or more protracted in nature, that differentially affects distribution, density and connectivity of neurons across cortical layers, could contribute to differences in cortical gyrification (and/or surface expansion) and would be detected in-vivo by macro-level scale morphological measures, such as the millimeter-scale gyrification index (i.e., sensitive to intrinsic curvature differences of cortical surface [35]) and FD complexity metrics as applied to cortical GM (e.g., [38]), or to subcortical GM, as we have shown, in schizophrenia. Note that here and in the next section we do not wish to make a strong claim about the underlying causes of observed FD reduction in the patient group, we merely suggest that a comprehensive attempt to link evidence available to date may do so across several levels of analysis. In this sense, our finding of fewer, less regularly distributed millimeter-level gyri in subcortical GM structures in schizophrenia patients is consistent with the micro-level evidence for differential expansion and/or altered neuronal density or distribution of cells in schizophrenia and, we suggest, may have important implications for our understanding of manifestations of schizophrenia.

### Significance of FD reduction in schizophrenia

Schizophrenia is a disorder of thought and, broadly speaking, represents a failure in the precision of predictions for the future [76]. From a high-level, computational account of schizophrenia, this disorder can be understood as a failure of metacognition—the inability of the brain to properly interpret causes of sensations coming from the various sensory systems to the brain [76]. These impairments may be linked to aberrant hippocampal morphology, since the hippocampus plays an important role in the binding of events that comprise human experience across space and time. We first note that about 90% of all cells in the hippocampal subfields CA1, CA2, and CA3 [77] constitute glutamatergic (excitatory) principal pyramidic neurons and are located in the central ‘pyramidale’ layer, with their corresponding cell parameters extending to “outer” layers, as follows. (Because hippocampus constitutes an independent fold of the cortical mantle, there is more than one “outer” layer closest to the surface being considered in the FD computation, in contrast to cortical GM where there is only one outer layer closest to the surface, that is, the upper layer containing dendrites). In particular, glutamatergic NMDA (N-methyl-D-aspartate) receptors (R) of these principal neurons are found on the apical dendrites (whose spines serve as postsynaptic input targets) in the ‘stratum radiatum’ layer (and to a lesser degree in the ‘lacunosum-moleculare’ layer), while the afferent and efferent axons and (inhibitory GABAergic) interneurons are located in ‘stratum oriens’ and ‘alveus’ layers [77].

Various lines of evidence support the role of NMDA-R dysfunction in schizophrenia. Empirical support comes from work showing that ketamine blocks NMDA receptors, reducing firing rates of neurons (e.g., [78]) and leading to trait abnormalities observed in schizophrenia patients such as cognitive impairments [76]. Further, experiments using genetic mouse models of schizophrenia demonstrate more specifically the role of NMDA-R in alterations in synaptic dysfunction (e.g., [79]). One possibility is that a less complex hippocampal surface may be associated with a reduced expansion or altered neuronal density and distribution of dendrites with NMDA-R, thus affecting post-synaptic gain. This is of interest for our investigation because trait abnormalities in schizophrenia may be associated with a relative decrease in the prior empirical precision of one’s beliefs, relative to sensory precision at a given moment [76], with “precision” thought to be encoded by the post-synaptic gain of pyramidal neurons [76]. In summary, aberrant structural morphology of the hippocampus, in particular reduction of its surface complexity quantified at millimeter-level scale, as we have shown here, may, we conjecture, contribute to aberrant beliefs and reasoning observed in schizophrenia [76] via possible irregularities in the post-synaptic gain of principal neurons (reflecting dysfunction in short-range connectivity) as well as further impact (longer-range) connectivity between brain networks [80] including aberrant structural [45] or functional connectivity with the pre-frontal cortex.

### Comparison to alternative subcortical shape analytic techniques

A recent shape analysis study found a surface inward-deformation in the anterior regions of bilateral hippocampi in patients with schizophrenia relative to healthy controls [45]. Although the directionality (i.e., reduction) of our findings is consistent with Qiu and colleagues [45], that study’s approach utilized a deformation map that references a hippocampal volume of each subject with respect to a template volume (i.e., thereby recording compressions, or inward-deformations and expansions, or outward-deformations). Crucially, that method requires smoothing of the input (which removes any surface abnormalities) prior to computation of potential deformations [49], meaning that some fine structural details may not be captured. Inconsistencies with respect to directionality of the deformations and their location along brain structures also exist when using this method (e.g., [44, 46, 47]). Furthermore, this method [44, 45, 47, 48] provides a qualitative description of where along the brain regions deformations were found, whereas FD is a quantitative approach that provides a compact numerical estimate associated with each GM structure for each patient and control participant. Therefore, although the capacity to identify where along the surface of a structure alterations exist (i.e., considering the differential topographical mapping of function in the hippocampus) may be argued to be advantageous over FD or conventional volume measurements, considering the above limitations of other currently used techniques, we suggest that FD approach can be used fruitfully when the goal is to examine subtle, pathophysiologically relevant, surface alterations of a given brain region, and to capture a single quantitative measure of surface complexity. In future work, FD metrics can also be adapted for hippocampal subfield analysis.

### Relationship to volume

Previous studies suggest that the differences in gray-matter volume may be partially mediated by surface anatomical properties such as thickness, surface area, and gyrification. Palaniyappan and Liddle [30], for example, found that gyrification index in the left insula and left temporal clusters mediated gray matter volumetric measures in patients with schizophrenia. Fractal analysis computations rely, as noted earlier, on both topological and non-topological aspects of the object. Because volume is a product of thickness and surface area, it is not surprising that FD output correlated with volume in both groups. If the two measures were fully dependent on each other, however, we would expect to find higher complexity in the presence of reduced volume. (We demonstrated that FD and volume measures are dissociable: decreasing volume does not increase FD; please see S2 Fig). We found that the shape of bilateral hippocampi and left thalamus was less complex in SCZ group relative to HC even though the volumes of these structures were reduced in schizophrenia patients (S1 Results). In addition, although we found that volume positively correlates with *D*_1_ measures, this association was similar for both diagnostic groups (S1 Results).

### Limitations & Future Directions

Complexity FD computations were performed for the entire GM surface of each subcortical structure. However, the FD value for a given brain structure in two individuals may be comprised of complexity contributions arising from different portions along this structure. With regard to hippocampal morphological and volumetric abnormalities in schizophrenia, previous work has found morphological deformations of the anterior (e.g., [43, 45]) well as inferior and superior [42] portions of the hippocampus. Reduced volume of the anterior region of the hippocampus has been reported [81], although some volumetric studies did not find region-specific abnormalities [82]. Sub-cortical structures are small, making detection of subtle surface differences computationally challenging. Nevertheless it would be interesting to investigate whether reduced complexity stems primarily from the anterior portion of the hippocampus, which contains populations of neurons that project to the prefrontal cortex (e.g., [83]). Examining the variations in complexity of certain portions within the subcortical structure, for example, by segmenting the anterior and posterior portions of the hippocampus using anatomical landmarks, is the goal of future work with larger case-control samples. An age by diagnosis interaction in right hippocampus and left thalamus revealed a negative association between FD and age in schizophrenia and will also need to be investigated in larger datasets, for example, using recently released open-access, publicly available datasets such as the MCIC Collection (http://coins.mrn.org).

Natural objects, unlike pure mathematical fractals, permit only an approximation of their FD. Moreover, our FD computations were made on the basis of MR images. However, although a limited voxel size resolution, a specific pre- or post-processing sequence, or noisy acquisition (cf. [59]) of MR images could in principle affect our FD estimates, we believe that their influence is minimal for the following reasons. All T1 images were collected on the same scanner (thus minimizing intensity differences across subjects) and were examined for potential presence of artifacts by author ZW; quality of all images was deemed suitable to enter the pre-processing pipeline. VBM8 was used for scull-stripping and bias-field correction and involved non-linear registration of images; while this step could introduce distortions, this was adjusted for automatically in VBM8 by using the ‘modulated normalize’ option. Moreover, the same pre-processing procedures were applied to T1 images of all participants. FD computations were conducted in standard MNI space by using output of fully automated segmentation procedures in FSL FIRST. We did not detect errors in the segmentation of GM structures by FSL FIRST by examining individual renderings for all participants. Overall, through the use of objective (automated) standard procedures, we attempted by minimize possible confounding variables during data processing so that any potential biases would not selectively or differentially impact between-group level inferences.

FD analytic technique produced highly variable output for very small structures (in particular, the nucleus accumbens). It is difficult to conjecture whether other shape analytic techniques may also produce more variable results because other investigators have typically focused on select subcortical regions such as the hippocampus and the thalamus. One possibility is that hippocampal formation may be especially susceptible to aberrant neurodevelopmental processes. Neonates at high risk for schizophrenia studied by Shi and colleagues, for example, have been found to have connectivity abnormalities, in particular, manifested as a less symmetrical hub distribution between left and right hippocampus [84]. It may be that the FD technique is not suitable for in-vivo analysis of very small subcortical structures (e.g., those below approximately 1,000 mm^3^). Therefore, even though subtle morphological differences between patients with schizophrenia and healthy controls may exist also in these other subcortical regions, here we show that FD technique is suitable to study, in-vivo, subtle morphological alterations of hippocampus and thalamus.

A current challenge in the field of computational anatomy is the lack of availability, in part for safety reasons, of large samples of patients with schizophrenia who are not prescribed antipsychotic medication (which has been noted to affect subcortical volumes). In particular, for GM subcortical structures, this question may be addressed in first-episode patients (with a duration of illness less than two years), and who have never taken antipsychotics or have had a history of substance abuse, including nicotine dependency. A finding of reduced complexity in non-medicated patients would lend additional support—as a brain trait—for the neurodevelopmental account of schizophrenia. If a significant difference in complexity is only found for those patients taking medication, then this information may nevertheless be informative as it would provide evidence that antipsychotics exert influence even on the subtle structural properties of the cortex as measured via fractal analysis.

## Conclusion

Captured using a quantitative FD metric, we find that irregularity of surface structure is informative and meaningful, revealing new features that characterize subcortical GM in individuals with schizophrenia relative to healthy controls. In this study we hypothesized that patients with schizophrenia have subtle structural irregularities in subcortical GM structures. The current work reports quantitative evidence for significant reduction in the complexity of surface structure, at the millimeter-level scale, of bilateral hippocampus and left thalamus in schizophrenia (lower FD; large Cohen’s effect sizes, U3). In summary, our preliminary findings demonstrate utility of fractal analysis in the quantitative assessment of morphological features of subcortical GM in schizophrenia.

## Supporting Information

S1 Fig Demonstration that an object with the same volume may have different FD outputs. An object with fixed volume but with different structural properties (e.g., objects in the right-handed column were divided into 22 and 10 lateral slices relative to the original cube) yields different FD outputs. Note: Cube image size: 110 x 110 x 110; RotatedCube(10) image size: 149 x 150 x 110; RotatedCube(22) image size: 149 x 150 x 110.
(TIF)

S2 Fig Demonstration that reducing volume of an object does not change FD outputs. Here we show that when a sphere made of 1.5 mm cubes (1.5 mm x 1.5 mm x 1.5 mm) with diameter 60 mm (radius = 30 mm) is compared to sphere made of 1.5 mm cubes with diameter 45 (radius = 22.5 mm), the FD output associated with increasingly smaller spheres is relatively constant around FD 2.95 (*r* = 0.18, *p* = 0.72), even though the volume (V = 4/3*pi*r^3) is reduced by about 50%. In particular, the volume is 113,100 for the largest sphere (diameter 60 mm) vs. 47,713 for the smallest sphere (diameter 45 mm) (i.e., a reduction of diameter by about 25%). This illustrates that a (linear) reduction in volume does not necessitate a comparable reduction (or increase) in the corresponding FD values. Note: d(60 mm diameter) image size: 80 x 80 x 80; d(57 mm) image size: 76 x 76 x 76; d(54 mm) image size: 72 x 72 x 72; d(51 mm) image size: 68 x 68 x 68; d(48 mm) image size: 64 x 64 x 64; d(45 mm) image size: 60 x 60 x 60.
(TIF)

S3 Fig The relative size (volume) of different subcortical GM structures relative to the standard deviation of the corresponding FD output. This figure illustrates that structures smaller by volume are associated with a larger standard deviation of FD output. Left panel illustrates the patient group (*r* = -0.6471, *p* = 0.0124); Right panel illustrates the healthy control group (*r* = -0.6236, *p* = 0.0172).
(TIF)

S4 Fig Illustrating reconstructions of subcortical structures. Left hippocampus, right hippocampus, and left thalamus are shown for a representative control participant (a) and a patient with schizophrenia (b).
(TIF)

S1 File Image Data. (TAR)

S2 File FD Data. (XLS)

S1 Results Association between FD and Volume. (DOCX)

S1 Table Volume measures for subcortical structures. (DOCX)

S1 Text Subcortical Structures. (DOCX)
