# Correction: Fractal Dimension Analysis of Subcortical Gray Matter Structures in Schizophrenia

**DOI:** 10.1371/journal.pone.0164910

**Published:** 2016-10-11

**Authors:** The PLOS ONE Staff

In the Author Contributions section, Guihu Zhao (GHZ) should be listed as one of the persons who analyzed the data. The publisher apologizes for the error.
