# Supplementary figures and images for: Fractal Dimension Analysis of Subcortical Gray Matter Structures in Schizophrenia
Source: PLoS One. 2016 May 13;11(5):e0155415. doi: 10.1371/journal.pone.0155415 (PMC4866699; doi:10.1371/journal.pone.0155415)

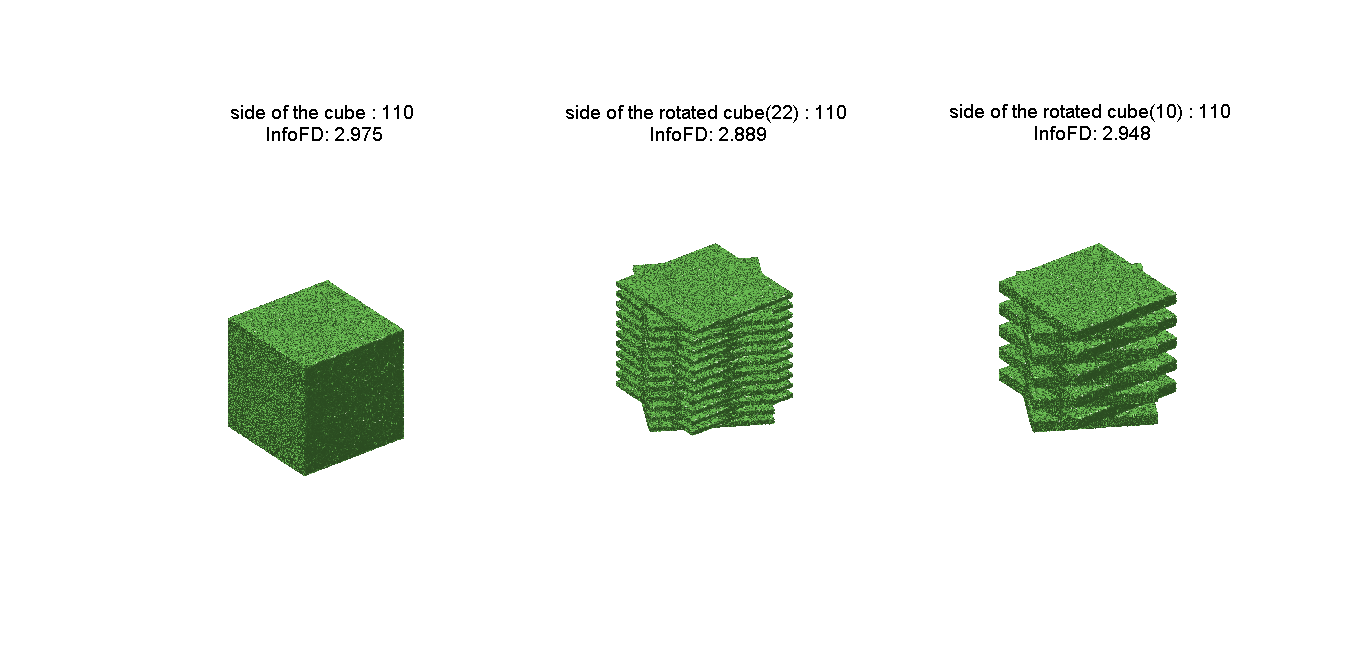

Supplement: S1 Fig — An object with fixed volume but with different structural properties (e.g., objects in the right-handed column were divided into 22 and 10 lateral slices relative to the original cube) yields different FD outputs. Note: Cube image size: 110 x 110 x 110; RotatedCube(10) image size: 149 x 150 x 110; RotatedCube(22) image size: 149 x 150 x 110. (TIF) [file pone.0155415.s001.tif]

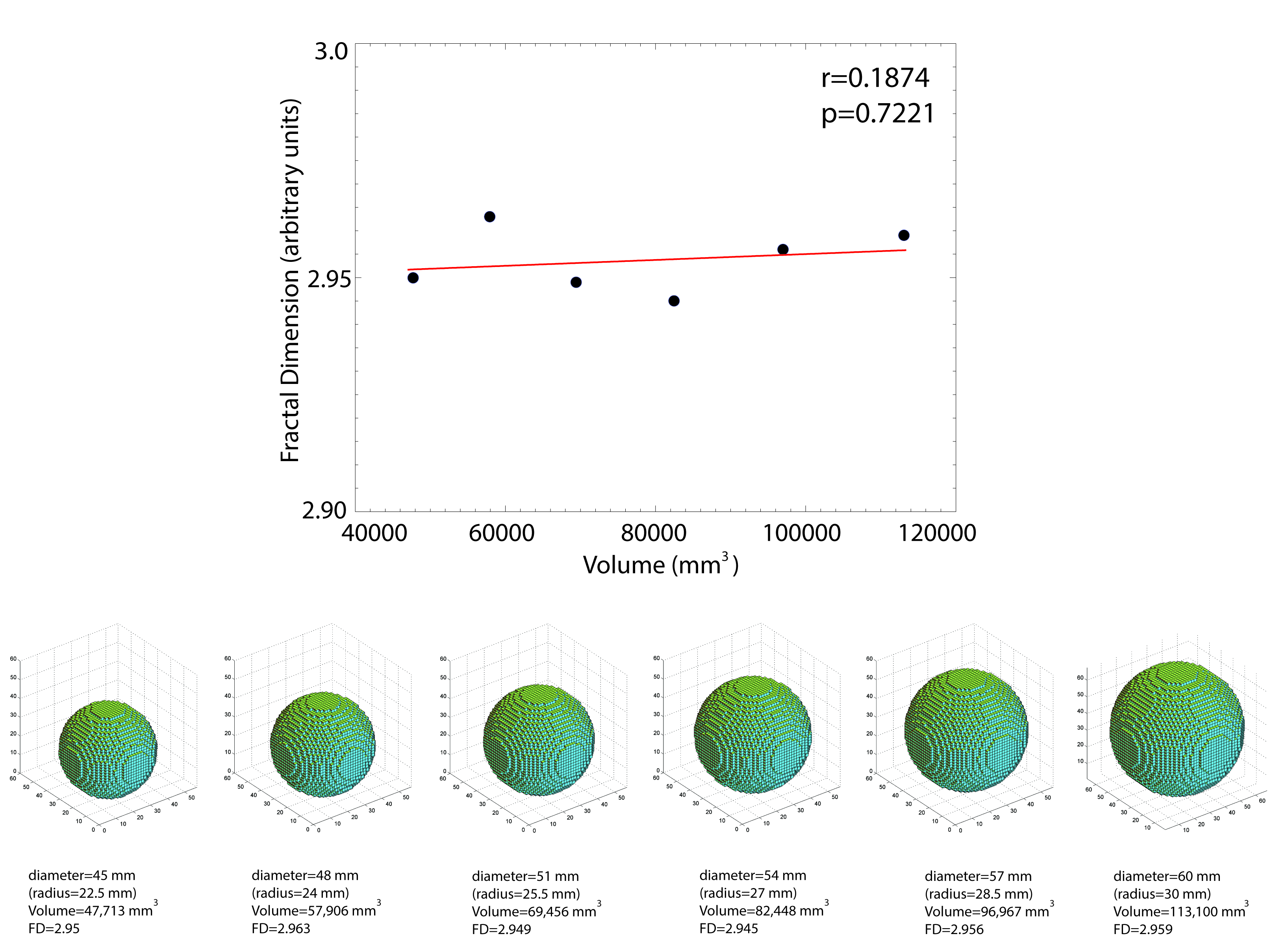

Supplement: S2 Fig — Here we show that when a sphere made of 1.5 mm cubes (1.5 mm x 1.5 mm x 1.5 mm) with diameter 60 mm (radius = 30 mm) is compared to sphere made of 1.5 mm cubes with diameter 45 (radius = 22.5 mm), the FD output associated with increasingly smaller spheres is relatively constant around FD 2.95 (r = 0.18, p = 0.72), even though the volume (V = 4/3*pi*r^3) is reduced by about 50%. In particular, the volume is 113,100 for the largest sphere (diameter 60 mm) vs. 47,713 for the smallest sphere (diameter 45 mm) (i.e., a reduction of diameter by about 25%). This illustrates that a (linear) reduction in volume does not necessitate a comparable reduction (or increase) in the corresponding FD values. Note: d(60 mm diameter) image size: 80 x 80 x 80; d(57 mm) image size: 76 x 76 x 76; d(54 mm) image size: 72 x 72 x 72; d(51 mm) image size: 68 x 68 x 68; d(48 mm) image size: 64 x 64 x 64; d(45 mm) image size: 60 x 60 x 60. (TIF) [file pone.0155415.s002.tif]

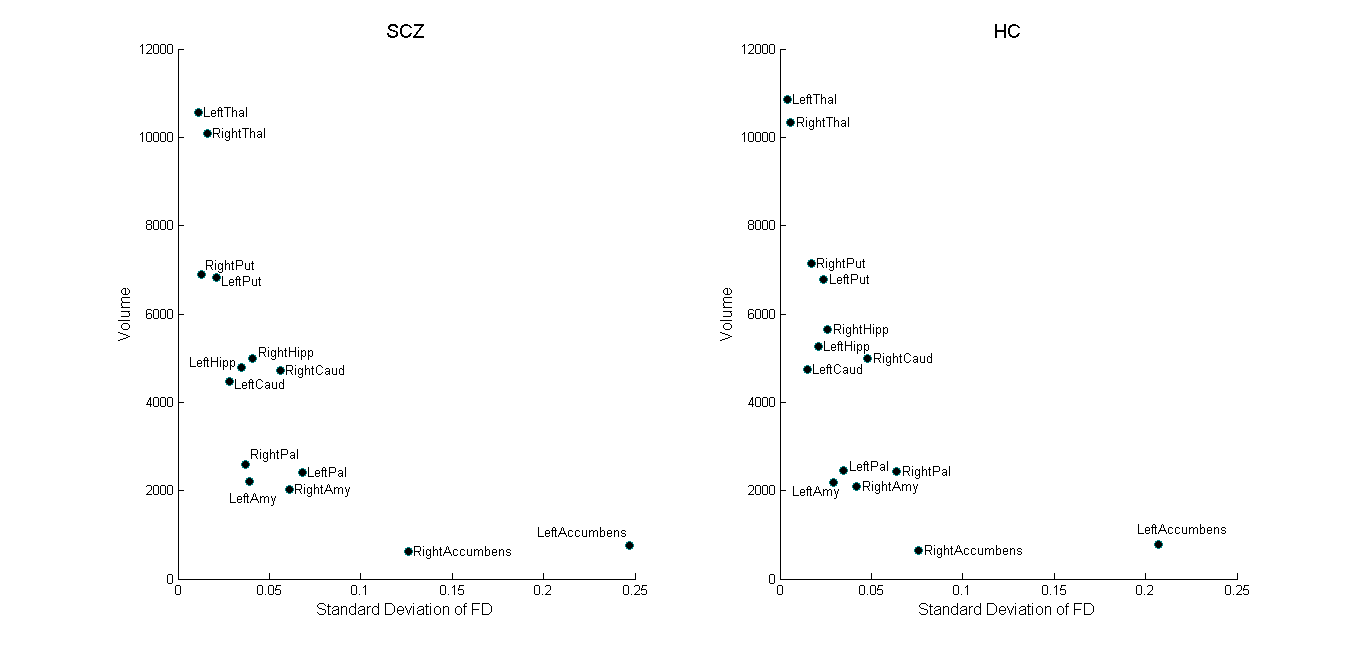

Supplement: S3 Fig — This figure illustrates that structures smaller by volume are associated with a larger standard deviation of FD output. Left panel illustrates the patient group (r = -0.6471, p = 0.0124); Right panel illustrates the healthy control group (r = -0.6236, p = 0.0172). (TIF) [file pone.0155415.s003.tif]

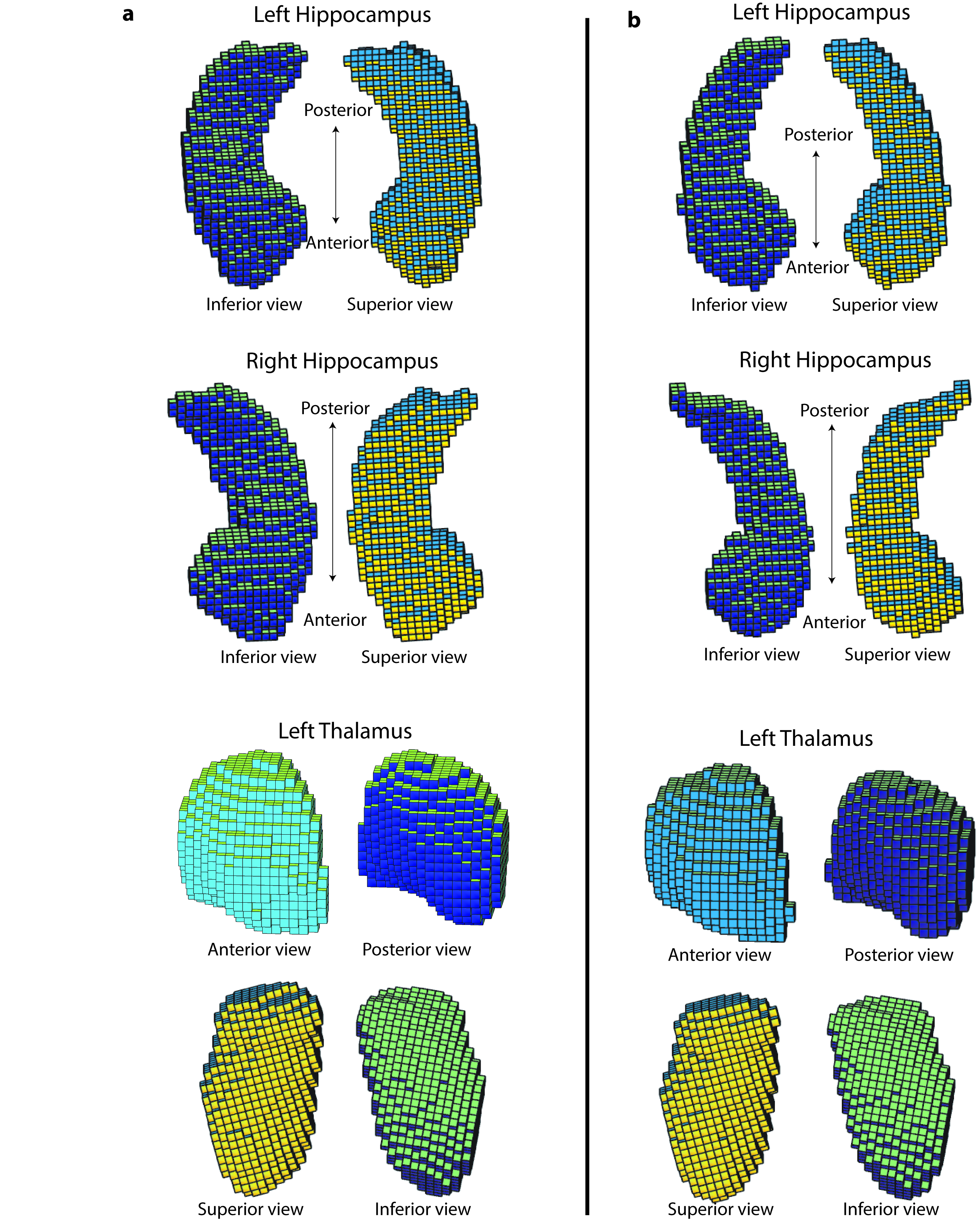

Supplement: S4 Fig — Left hippocampus, right hippocampus, and left thalamus are shown for a representative control participant (a) and a patient with schizophrenia (b). (TIF) [file pone.0155415.s004.tif]
