# Supplementary material for: Fractal Dimension Analysis of Subcortical Gray Matter Structures in Schizophrenia
Source: PLoS One. 2016 May 13;11(5):e0155415. doi: 10.1371/journal.pone.0155415 (PMC4866699; doi:10.1371/journal.pone.0155415)
Supplement: S1 Results — (DOCX) [file pone.0155415.s007.docx]

## **Association between FD and volume**

We sought to examine the association between *D*_1_ and volume for the three subcortical GM structures for which we found a significant difference between SCZ and HC. The association was tested using both local volumes (mm^3^) values (covarying for the total subcortical volume including the brainstem using general linear model) as well as using the residuals of the expected (predicted) local volumes given the total subcortical volume (Arndt et al., 1991). Specifically, residual value is the difference (least squares) between the structure’s measured volume (Y) and the predicted value (Y’): Y-Y’ (here, on the basis of the total subcortical volume which includes the brainstem); the residuals are derived using linear regression in SPSS. Unstandardized residuals were used. Correlation coefficients were also computed to test the association between complexity (*D*_1_) outcomes and volume using rank-test (Spearman's *rho*) for left and right hippocampi and left thalamus (significance set to p<0.05).

Volumes were reduced in the SCZ group relative to HC for left hippocampus: SCZ: 4777.401 ± 112.325, HC: 5262.336 ± 91.318, *p* = 0.003, right hippocampus: SCZ: 4999.796 ± 137.909, HC: 5646.730 ± 90.433, *p* = 0.001, and left thalamus SCZ: 10559.842 ± 112.373, HC: 10864.836 ± 41.394, *p* = 0.022 (values indicate mean volume in mm^3^ and corresponding standard deviation). (Volume measures for all structures are listed in S1 Table).

We found that *D*_1_ outcomes correlated with the corresponding volume for each structure for both groups using all three methods. When using a rank-test, correlation coefficients between *D*_1_ and the corresponding volume (Spearman's *rho*) were: left hippocampus SCZ: *rho* = 0.921, HC: *rho*=0.891, right hippocampus SCZ: *rho*=0. 868, HC: *rho* = 0.807, and left thalamus SCZ: *rho*= 0.903, HC: *rho*= 0.761 (all p < 0.001). When covarying for total subcortical volume (including the brainstem), correlation coefficients between *D*_1_ and the corresponding volume were (Pearson’s *r*): left hippocampus SCZ: *r* = 0.93, HC: *r* = 0.87, right hippocampus SCZ: *r* = 0.901, HC: *r* = 0.802, and left thalamus SCZ: *r* = 0.744, HC: *r* = 0.833 (all *p* < 0.001). When using residuals, correlation coefficients between *D*_1_ and the corresponding residuals were also positive: left hippocampus SCZ: *r* = 0.93, HC: *r* = 0.87, right hippocampus SCZ: *r* = 0.853, HC: *r* = 0.798, and left thalamus SCZ: *r* = 0.962, HC: *r* = 0.792 (all *p* < 0.001).
