# Supplementary material for: Fractal Dimension Analysis of Subcortical Gray Matter Structures in Schizophrenia
Source: PLoS One. 2016 May 13;11(5):e0155415. doi: 10.1371/journal.pone.0155415 (PMC4866699; doi:10.1371/journal.pone.0155415)
Supplement: S1 Table — (DOCX) [file pone.0155415.s008.docx]

**S1 Table. Volume measures for subcortical structures**

|  |  |  |  | Left |  | | Right |  |  | | |
| --- | --- | --- | --- | --- | --- | --- | --- | --- | --- | --- | --- |
| Structure |  | mean ± sd | sem | median | min | max | mean ± sd | sem | median | min | max |
| Thalamus | SCZ | 10559.842±489.824 | 112.373 | 10668.375 | 9311.625 | 11178.000 | 10097.112±493.850 | 113.297 | 10108.125 | 9028.125 | 10951.875 |
|  | HC | 10864.836±180.434 | 41.394 | 10850.625 | 10476.000 | 11238.750 | 10345.441±201.058 | 46.126 | 10300.500 | 10000.125 | 10725.750 |
| Caudate | SCZ | 4472.408±484.404 | 111.13 | 4529.250 | 3520.125 | 5450.625 | 4729.618±484.000 | 111.037 | 4826.250 | 3631.500 | 5396.625 |
|  | HC | 4733.526±305.862 | 70.17 | 4731.750 | 4191.750 | 5241.375 | 5003.349±434.398 | 99.658 | 5018.625 | 4336.875 | 5859.000 |
| Putamen | SCZ | 6820.697±361.294 | 82.887 | 6888.375 | 6162.750 | 7290.000 | 6886.243±320.147 | 73.447 | 6976.125 | 6237.000 | 7519.500 |
|  | HC | 6791.033±375.825 | 86.22 | 6864.750 | 6169.500 | 7489.125 | 7148.783±274.083 | 62.879 | 7188.750 | 6483.375 | 7587.000 |
| Palllidum | SCZ | 2401.224±410.983 | 94.286 | 2490.750 | 1019.250 | 2895.750 | 2593.599±173.576 | 39.821 | 2605.500 | 2325.375 | 3010.500 |
|  | HC | 2450.428±252.981 | 58.038 | 2409.750 | 2153.250 | 3148.875 | 2432.487±340.936 | 78.216 | 2473.875 | 1225.125 | 2824.875 |
| Hippocampus | SCZ | 4777.401±489.613 | 112.325 | 4782.375 | 3803.625 | 5649.750 | 4999.796±601.129 | 137.909 | 5167.125 | 3705.750 | 5737.500 |
|  | HC | 5262.336±398.044 | 91.318 | 5244.750 | 4482.000 | 5926.500 | 5646.73±394.188 | 90.433 | 5666.625 | 4968.000 | 6365.250 |
| Amygdala | SCZ | 2217.375±134.700 | 30.902 | 2224.125 | 2004.750 | 2558.250 | 2027.132±211.009 | 48.409 | 2035.125 | 1650.375 | 2413.125 |
|  | HC | 2175.276±259.971 | 59.641 | 2149.875 | 1869.750 | 2669.625 | 2096.23±208.210 | 47.767 | 2109.375 | 1704.375 | 2662.875 |
| Nucleus  Accumbens | SCZ | 764.704±125.377 | 28.764 | 762.750 | 499.500 | 1073.250 | 631.658±120.255 | 27.588 | 600.750 | 475.875 | 921.375 |
|  | HC | 791.882±94.441 | 21.666 | 806.625 | 631.125 | 968.625 | 643.559±98.962 | 22.703 | 641.250 | 499.500 | 830.250 |
| Brain Stem | SCZ | 32693.803±21771.534 | 4994.732 | 26942.625 | 24931.125 | 121763.250 |  |  |  |  |  |
|  | HC | 27143.704±2133.718 | 489.509 | 26939.250 | 24934.500 | 35312.625 |  |  |  |  |  |

Volume measures for the seven subcortical GM structures, bilaterally, and the brain stem, for schizophrenia patients (SCZ) and healthy controls (HC). The values denote mean, standard deviation, standard error of the mean (s.e.m., within-group), median, range (minimum and maximum).
