# Supplementary material for: Fractal Dimension Analysis of Subcortical Gray Matter Structures in Schizophrenia
Source: PLoS One. 2016 May 13;11(5):e0155415. doi: 10.1371/journal.pone.0155415 (PMC4866699; doi:10.1371/journal.pone.0155415)
Supplement: S1 Text — (DOCX) [file pone.0155415.s009.docx]

## **Subcortical structures**

*Hippocampus* is a “highly convoluted” and evolutionarily older structure compared to the neocortex [[1](#_ENREF_1)], with the laminated (layered) architecture characteristic of GM folds of the cortical mantle [[2](#_ENREF_2)]. Incoming projections to the hippocampus arrive from the cortex via entorhinal cortex (EC) layers II and III [[2](#_ENREF_2)], while the output arises from hippocampal subfield CA1 (cornu ammonis) and is projected out to the parahippocampal formation as well as to the deep layers of entorhinal cortex (EC) [[3](#_ENREF_3)]; the EC together with the hippocampus proper comprise the hippocampal formation. The deep layers of EC project information back to the cortex. The *caudate*, the *putamen*, and the *pallidum* are part of the basal ganglia, a subcortical loop linking the cerebral cortex with upper motor neurons in the primary motor and premotor cortex and the brainstem [[1](#_ENREF_1)]. The caudate and the putamen receive inputs from the cortex via medium spiny neurons that have large dendritic trees (a property which facilitates integration of neural signals) from the cortex, thalamus, and the brainstem, while the outgoing axons from these structures converge in the pallidum. The efferent neurons in the pallidum send information to motor cortex and the brainstem [[1](#_ENREF_1)]. The *nucleus accumbens* contains medium spiny neurons that integrate excitatory signals under the modulatory influence of dopamine; this structure receives signals relevant to emotion processing [[1](#_ENREF_1)]. The t*halamus* receives peripheral sensory information and has neurons that project to primary sensory cortices [[1](#_ENREF_1)] as well as to the dorso-lateral pre-frontal cortex [[4](#_ENREF_4)]. The *amygdala* is part of the limbic system, with projections to the pre-frontal cortex, the cortical areas that process sensory information as well as to the thalamic nuclei and the brainstem [[1](#_ENREF_1)]. The *brainstem* contains both ascending and descending sensory nerves from the head and neck and the descending motor tracks from the forebrain [[1](#_ENREF_1)].

**References**

1. Purves, D., Augustine, G.A., Fitzpatrick, D., Hall, W., LaMantia, A-S., McNamara, J.O., & White, L. Neuroscience. 4th ed: Sunderland, MA: Sinauer Associates.

2. Vida I. Morphology of hippocampal neurons. In: Cutsuridis, V, Graham, B, Cobb, S, Vida, I, editors Hippocampal microcircuits: A computational modeler’s resource book. New York: Springer; 2010. p. 27-67.

3. Van Strien N, Cappaert N, Witter M. The anatomy of memory: an interactive overview of the parahippocampal–hippocampal network. Nature Reviews Neuroscience. 2009;10(4):272-82.

4. Harrison PJ, Lewis, D. A. and Kleinman, J. E. . Neuropathology of Schizophrenia. In: Daniel R. Weinberger PJH, editor. Schizophrenia. 3rd ed: Wiley-Blackwell, Oxford, UK. ; 2011. p. 372-92.
